# Optimizing Emergency Response in Hospitals: A Systematic Review of Surge Capacity Planning and Crisis Resource Management

**DOI:** 10.3390/healthcare13212819

**Published:** 2025-11-06

**Authors:** Savvas Petanidis, Krishna Chandramouli, George Floros, Sokratis Nifakos, Kostas Kolomvatsos, Sofia Tsekeridou, Sabina Magalini, Daniele Gui, Christoforos Kosmidis

**Affiliations:** 1 School of Medicine, AHEPA Hospital, Aristotle University of Thessaloniki, 55236 Thessaloniki, Greece; gefloros@ieee.org (G.F.); kosmidisc@auth.gr (C.K.); 2 Rinicom Limited, Lancaster LA1 2RX, UK; krishna.chandramouli@rinicom.com; 3 Department of Electronic and Electrical Engineering, Trinity College Dublin, D02 PN40 Dublin, Ireland; 4 Massive Dynamic Sweden AB, 11349 Stockholm, Sweden; sokratis@massivedynamic.se; 5 Department of Informatics and Telecommunications, University of Thessaly, 35100 Lamia, Greece; kostasks@uth.gr; 6 Netcompany SA, 1253 Luxembourg, Luxembourg; sofia.tsekeridou@netcompany.com; 7 Fondazione Policlinico Universitario Agostino Gemelli IRCCS, Universita Cattolica Del Sacro Cuore, 00168 Rome, Italy; sabina.magalini@unicatt.it (S.M.); daniele.gui@unicatt.it (D.G.)

**Keywords:** hospital capacity planning, resource allocation, workforce management, healthcare preparedness, surge response strategies, patient surge capacity management, healthcare demand forecasting, resource planning, healthcare operational resilience

## Abstract

**Background**: Healthcare systems worldwide face growing challenges in anticipating and managing patient surges, particularly in times of public health crises, natural disasters, or seasonal peaks. The ability of healthcare organisations to forecast and respond to such demand fluctuations—referred to as organisational readiness for patient capacity surge—has become a critical determinant of service continuity and patient outcomes. Despite the urgency, there remains a lack of consolidated evidence on how healthcare authorities measure, evaluate, and operationalise this readiness. This systematic review aims to identify and synthesise existing literature that presents case studies, methodologies, and strategic frameworks used to evaluate organisational preparedness for patient surge capacity. It also explores resource allocation mechanisms, hospital capacity planning algorithms, and temporary facility strategies documented in healthcare settings. **Methods**: The review was conducted across two major scientific repositories, i.e., PubMed and Web of Science (WoS). A set of four structured search queries were formulated to capture the breadth of the topic, focusing on demand forecasting, hospital capacity planning, workforce models, and resource management within the context of healthcare surge demand. The search was limited to publications from the last 10 years (2014–2024) to ensure the inclusion of contemporary practices and technologies. **Results**: A total of 142 articles were selected for detailed analysis. The articles were categorised into six thematic groups: (i) empirical case studies on healthcare surge management; (ii) hospital resources and capacity scaling; (iii) ethical frameworks guiding surge response; (iv) IT-driven algorithms and forecasting tools; (v) policy evaluations and actionable lessons learned; and (vi) existing systematic reviews in related domains. Notably, several articles provided evidence-based frameworks and simulation models supporting predictive planning, while others highlighted real-world implementation of temporary care facilities and staff redeployment protocols. **Conclusions:** The review underscores the fragmented yet growing body of literature addressing the multidimensional nature of surge preparedness in healthcare. While algorithmic forecasting and capacity modelling are advancing, gaps remain in standardising metrics for organisational readiness and incorporating ethical considerations in surge planning. Limitations of this review include potential selection bias and the subjective categorisation of articles. Future research should aim to develop integrative frameworks that couple technical, operational, and ethical readiness for patient surge scenarios.

## 1. Introduction

Around the world, emergency medical treatment is an essential part of healthcare systems [1]. However, emergency departments and hospitals face tremendous hurdles at times of hospital rush, such as the COVID-19 pandemic, natural disasters, or mass casualty events [2]. The unexpected increase in patient volume surpasses typical capacity, necessitating quick adjustments to staffing levels, procedures, and resource allocation in order to provide timely care [3]. This article examines the various approaches commonly adopted to control emergency medical care amid hospital overcrowding, highlighting the knowledge gained from previous lessons while proposing innovative lessons [4]. Disasters and emergencies can disrupt social and organizational operations; this disruption may surpass the healthcare provider’s ability to handle patient needs resulting in financial losses [5]. However, anticipating the issues that will arise from these occurrences and making plans to deal with them successfully are essential to managing these destructive and detrimental events efficiently. People’s health and well-being are their first priority during catastrophic disasters; hence, health systems are essential to reduce the number of fatalities and injuries [6]. The primary program for disaster management at the national level, particularly in nations that are prone to disasters, is the international motto of preparedness for handling emergencies and disasters, particularly hospital preparedness. Health systems and hospitals, in particular, are focused on strengthening and planning for surge capacity, which is the ability to offer medical and healthcare amid an abrupt increase in the number of patients or victims of emergencies and disasters [7].

More specifically, planning for surge capacity effectively is essential to preventing healthcare facilities from becoming overloaded [8]. Creating overflow wards, increasing the number of intensive care unit beds, and anticipating the need for additional staffing sources, such volunteers or retired medical personnel, are all part of the emergency response plans that hospitals should create and update on a regular basis. Disaster drills and simulation exercises are essential for preparing employees for real-world situations [9]. Internal research has shown that healthcare organizations can effectively implement the theory of limitations to address capacity management issues, shorten inpatient stays, and boost patient satisfaction with service delivery [10]. A surge may involve four different “S” domains of hospital service capacity—staffing, supplies, space, and systems—and it may be short-lived or long-lasting, lasting days, weeks, or months [11]. The effects of a medical surge on healthcare delivery can be found on a spectrum, from crisis management to contingency planning to conventional treatment. Hospitals need to have procedures and protocols in place in order to be able to care for patients who present, regardless of whether they are “ready” for accidents to happen [12].

Following the growing body of literature on hospital emergency management, particularly surge capacity, disaster preparedness, and resource optimization, there is a pressing need to consolidate research findings and identify best practices for mitigating overcrowding during crises. Since the COVID-19 pandemic, numerous studies have examined hospital responses to surges in patient volume, resource shortages, and systemic inefficiencies [7]. Several systematic reviews have previously examined hospital surge capacity, though most have concentrated on a single dimension, such as pandemic-specific surge responses, intensive care expansion, or ethical triage protocols [7,13]. By contrast, few reviews have attempted to integrate these diverse strands of evidence into a holistic synthesis. The present review addresses this gap by combining evidence across technical, operational, ethical, and policy domains, thereby offering a broader and more integrative perspective on hospital surge preparedness. The primary objective of this systematic review is to synthesize evidence from case studies research on hospital resources, IT algorithms, as well as ethical studies and policy evaluations to assess the effectiveness of various emergency management strategies in healthcare settings. To achieve this, we analyze a comprehensive collection of articles spanning from 2014 to 2024, focusing on how hospitals adapt to surges caused by pandemics, natural disasters, and mass casualty incidents.

The contributions of this paper include the following:

- Conducting an in-depth review of surge capacity strategies employed by hospitals during high-stress scenarios, including the expansion of physical space, staffing adjustments, and supply chain resilience.
- Evaluating the role of IT algorithms in improving staff readiness and operational efficiency during emergencies.
- Assessing the impact of hospital policies on reducing patient wait times and optimizing resource allocation.
- Examining case studies of hospitals that successfully (or unsuccessfully) managed surge events, identifying key lessons for future preparedness.
- Reviewing international frameworks and guidelines for emergency response in healthcare systems, highlighting gaps and opportunities for standardization.

The rest of the paper is structured as follows. Section 2 outlines the Preferred Reporting Items for Systematic Reviews and Meta-Analyses (PRISMA) methodology used to select and analyze relevant studies. Section 3 presents the findings, categorizing them by themes such as staffing models, infrastructure adaptations, IT algorithms, and systemic challenges. Section 4 discusses policy interventions and lessons learned. Section 5 discusses emerging strategies, including technological innovations, while also identifying areas requiring further research. Finally, Section 6 summarizes key conclusions and proposes future directions for improving hospital emergency management.

## 2. Materials and Methods

This section outlines the systematic methodology employed for this review, structured in accordance with the PRISMA guidelines [14]. The process comprised based on the following key phases:

### 2.1. Research Questions

The scope of emergency management in hospitals spans operational, technological, and ethical dimensions, encompassing (i) physical resource allocation during surges, (ii) AI/ML-driven decision-support systems, (iii) ethical frameworks for crisis care, and (iv) policy evaluations for systemic resilience. To systematically investigate these interconnected domains, we formulated five targeted research questions examining demand forecasting models, algorithmic surge prediction tools, ethical challenges in rationing, and policy effectiveness, as shown in Table 1. Each question was designed to identify critical challenges healthcare institutions face when preparing for and responding to patient surges, with particular attention to studies involving multidisciplinary hospital staff (administrators, clinicians, emergency planners, and support personnel). To ensure comprehensive coverage of relevant literature, targeted search strings were developed and applied across major bibliographic databases.

### 2.2. Protocol and Eligibility Criteria

As we mentioned earlier, the systematic review adheres to the PRISMA framework [14]. We included peer-reviewed articles published between 2014–2024 that specifically examined hospital emergency management in clinical settings (emergency departments, ICUs) and healthcare systems responding to surges (pandemics, natural disasters, mass casualty events). The review focuses on peer-evaluated evidence across four critical domains, i.e., surge capacity planning, resource allocation algorithms, ethical challenges in crisis care, and policy effectiveness. Eligible studies were required to present empirical data from hospital operations and address at least one of our predefined research questions (Table 1). We excluded non-peer-reviewed publications, theoretical studies, and pre-hospital care research without hospital application. The selection prioritized multidisciplinary evidence encompassing frontline clinicians, hospital administrators, and health policymakers to evaluate organizational resilience during surge events.

In addition, this review focused on PubMed and Web of Science (WoS) as primary data sources. These two repositories were chosen due to their broad coverage of biomedical, clinical, and interdisciplinary health system research, which aligns closely with the scope of our research questions. Preliminary scoping searches in other databases (e.g., Cochrane Library, EMBASE, CINAHL) demonstrated substantial overlap with studies already identified in PubMed and WoS, supporting our decision to avoid redundancy and maintain methodological efficiency. In line with PRISMA guidelines, we prioritized peer-reviewed evidence to ensure transparency and reproducibility. Grey literature, although potentially informative, was excluded to focus on empirically validated studies and maintain comparability across included works.

### 2.3. Information Sources

The systematic search was executed across PubMed and WoS using four targeted search strings aligned with our research questions on healthcare demand forecasting and surge capacity (2014–2024). Search results from both databases were exported to EndNote for removing duplicates and collaborative screening, where the research team applied inclusion/exclusion criteria through a three-stage process (Include/Exclude/Maybe). All ‘Maybe’ articles underwent dual independent review with conflicts resolved through consensus. The initial 392 identified records were reduced to 369 after deduplication and title/abstract screening, with 142 studies ultimately selected for full-text review. This PRISMA-compliant process was documented in EndNote through shared libraries, custom tags were assigned to each of the publication under consideration by individual assessors. The independent analysis was shared among the assessors for enabling final decisions. When a conflict was raised among the assessors, a discussion on the publication was held that paved the pathway to resolve the conflict. Subsequently, annotated PDFs with rationales were shared, ensuring transparent and reproducible study selection.

### 2.4. Searches

We conducted a comprehensive literature search across multiple databases to identify relevant studies on healthcare demand forecasting and surge capacity management. Our search strategy comprised four primary queries, executed as follows:

Queries 1–4 were run in both PubMed and Web of Science (WoS) to ensure broad coverage The specific search queries, designed to address our four research questions, were:

- Q1: Search: (((healthcare) OR (medical service)) AND ((demand forecasting)) OR (demand estimation)), Filters: 2014–2024, English.
- Q2: ((((healthcare) OR (medical service)) AND (algorithm forecast surge) OR (hospital capacity planning) OR (resource allocation) OR (surge demand))) OR (resource allocation), Filters: 2014–2024, English.
- Q3: ((staffing models) OR (workforce management)) AND (healthcare surge demand), Filters: 2014–2024, English.
- Q4: ((hospital capacity management) AND (temporary facilities)) AND (resource management), Filters: 2014–2024, English.

After removing duplicates, we identified 369 potentially relevant articles. Through rigorous screening using the PRISMA-guided filtering process, we ultimately selected 142 studies for inclusion in this systematic review. The selection process emphasized studies with direct relevance to hospital emergency management, surge capacity planning, and healthcare demand forecasting methodologies.

### 2.5. Inclusion and Exclusion Criteria

We adopted a hierarchical application of inclusion/exclusion criteria in the filtering process. During the first stage, the research team unanimously agreed that each article should be screened against generic criteria defining the scope of the systematic review. The following criteria were used to identify relevant studies:

- Articles addressing hospital emergency management strategies during surges (e.g., pandemics, natural disasters, mass casualty events).
- Studies evaluating surge capacity adaptations, such as staffing models, overflow wards, or ICU bed expansion.
- Research on emergency preparedness frameworks and IT algorithms, including simulation exercises, policy interventions, or resource allocation protocols.
- Case studies reporting lessons learned from real-world hospital crises, with emphasis on systemic challenges or successful mitigation efforts especially during the COVID-19 period.
- Articles discussing international or national guidelines for hospital emergency response.

Irrelevant studies were filtered out using the following criteria:

- Studies not aligned with the research questions (e.g., non-hospital settings, non-emergency contexts).
- Articles written in languages other than English.
- Duplicate publications or overlapping datasets.
- Studies focused solely on pre-hospital emergency services (e.g., EMS, ambulance logistics) without hospital integration.
- Research exclusively on public health surveillance or epidemiological modeling without direct ties to hospital management.

**Figure 1:**
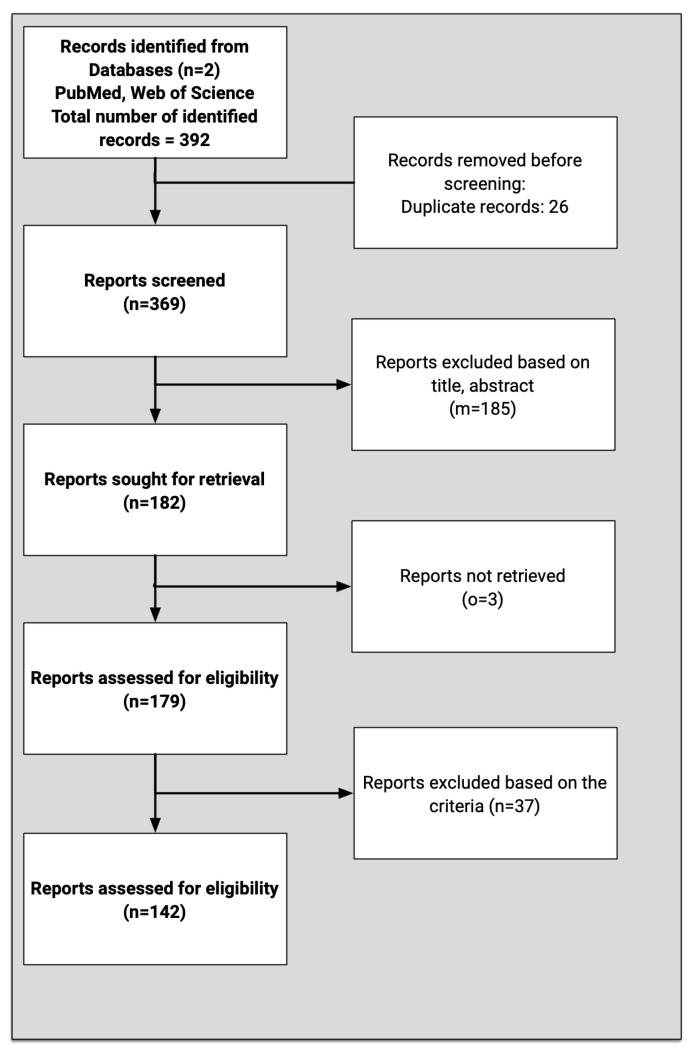
Literature search process, according to PRISMA recommendations.

To systematically manage the screening process, articles were categorized into three groups: ‘Include’ (directly relevant to the review’s objectives), ‘Exclude’ (not meeting the inclusion criteria), and ‘Maybe’ (requiring additional evaluation). The “Maybe” category articles were independently assessed by two researchers, with consensus required for final inclusion. This rigorous selection process narrowed the initial 392 identified papers down to 142 highly relevant articles for detailed examination in subsequent phases of the review. The overall approach adopted based on the PRISMA recommendation is presented in Figure 1.

### 2.6. Article Selection

After selecting the 142 articles, we implemented a rigorous relevance assessment process where each study was independently evaluated by two researchers against our four research questions (demand forecasting, capacity planning, staffing models, and temporary facility management) using a 5-point scoring system. Scores were averaged when researcher agreement was within 5% deviation and cases with greater discrepancies were resolved through consensus discussions. This approach allowed us to objectively quantify each article’s contribution while accounting for interdisciplinary overlaps in emergency management research. The scoring methodology not only ensured consistency in selection but also revealed important thematic connections across different aspects of hospital emergency preparedness. Each article was assessed against predefined criteria using a 5-point scoring system to capture relevance to the research questions and methodological rigor. Two independent reviewers performed the scoring, and discrepancies were resolved through discussion. Inter-observer agreement exceeded 90%, underscoring the robustness of the selection process. The scoring framework is summarized in Table 2.

Due to the heterogeneity of study designs, populations, and outcome measures across the included studies, we employed a descriptive synthesis rather than attempting a meta-analysis. While formal pooling of effect sizes was not feasible, we have indicated the distribution of study types and thematic coverage to enhance transparency and comparability across domains.

### 2.7. Data Extraction

Throughout the systematic review process, all references and screening data were systematically organized using EndNote. EndNote served as a centralized platform for collaborative reference management, enabling the research team to efficiently track and evaluate articles at each stage of the review. While multiple tools exist for managing scholarly literature, EndNote provided a structured workflow for storing full-text articles in PDF format, annotating key findings, and documenting decisions on inclusion or exclusion. Each researcher independently annotated articles to assess their relevance to the research objectives, with these annotations serving as documented evidence to support the final selection. Collaborative discussions among researchers were instrumental in resolving uncertainties, particularly for articles requiring deeper scrutiny. Special attention was given to debates surrounding the inclusion of large-scale case studies (e.g., national-level emergency response evaluations), which were critically examined for their applicability to the review’s focus on hospital surge capacity and disaster preparedness. These deliberations enhanced the rigor and coherence of the final article selection.

## 3. Results

Although the six themes are presented separately, several overlaps were evident. For example, IT-driven forecasting models were often embedded within broader staffing strategies, while ethical frameworks were consistently referenced in shaping policy implementation. These cross-thematic insights highlight that surge preparedness is not confined to isolated strategies but rather emerges from the interplay of technical, operational, and ethical dimensions.

### 3.1. Hospital Resources

Health systems globally face challenges in balancing equity and efficiency when allocating hospital resources. Studies across Chinese provinces [15,16,17,18] employ metrics like the Theil index and Gini coefficient to reveal persistent disparities, with urban centers like Chengdu exhibiting better geographic equity than rural regions such as Panxi. Research in Jilin Province [19] and elderly care institutions [20] further highlights how demographic shifts exacerbate inequities, while [21] proposes a multi-criteria framework incorporating societal values into allocation decisions. Cross-country comparisons in Europe [22,23,24] and analyses of medical alliances [25] demonstrate how hierarchical systems can either mitigate or amplify these gaps. Methodological advances, including three-stage DEA [17] and coupling coordination models [20,26], provide tools to optimize bed and staff distribution while accounting for regional economic differences. Also, ref. [27] introduces portfolio uncertainty modeling for risk-averse decision-making in resource allocation, particularly relevant for budget-constrained settings. Finally, in ref. [28] a spatiotemporal EMS demand forecasting model informs equitable ambulance distribution across urban/rural areas, while [29,30] evaluates forecasting methods for emergency resource allocation.

The COVID-19 pandemic drove several innovations in predictive modeling for hospital demand. Machine learning approaches such as BILSTM-GASVR [31] and SARIMAX-LSTM hybrids [32,33] outperformed traditional statistical methods in forecasting ICU needs and blood component demand. Ensemble models combining epidemiological data with mobility metrics [34,35] and near-real-time dashboards [36,37] improved short-term predictions for emergency departments. Studies in New Zealand [38,39], Italy [40,41], and Germany [42,43,44] demonstrated the value of adaptive models that incorporate local transmission dynamics and variant-specific parameters. In addition, ref. [45] provided a time-series study of Milan EDs that demonstrated how incorporating meteorological data improved visit forecasting accuracy. For longer-term planning, grey system theory [19] and discrete-time Markov chains [46,47] helped hospitals anticipate resource needs during multi-wave outbreaks. However, challenges persist in accounting for sudden demand surges [48,49] and predicting post-pandemic recovery of elective care volumes [50,51].

Global experiences during COVID-19 revealed critical lessons in surge capacity management. The ARRAS framework [52] documented workforce retention strategies in South Asia, while Australian ICUs [53,54] and U.S. hospitals [55,56,57] shared protocols for rapid bed expansion and staff redeployment. In Swedish hospitals, the dynamic balance of COVID/non-COVID demand through real-time capacity dashboards was proposed in [58]. Team-based care models [54,59,60] and telehealth integration [48] helped maintain quality during staff shortages. Case studies from field hospitals [61,62] and disaster zones [63,64,65] emphasized the importance of modular design and supply chain resilience. However, systematic reviews of H1N1 responses [13] warned that surge strategies often neglect patient safety metrics, highlighting the need for standardized performance monitoring during crises [60,66,67].

Moreover, discrete-event simulation emerged as a powerful tool for hospital resource management. Models balancing COVID-19 and elective care [68,69,70] identified optimal ICU “ring-fencing” thresholds, while queueing theory applications [71] quantified the impact of ambulance diversion policies. Integrated location-allocation models [46,72,73] improved disaster response planning for mass casualty events, and hybrid optimization-simulation approaches [74,75] reduced ED wait times by 44%. Real-time location systems [75] enabled dynamic staff scheduling, and microsimulations of patient flow [76,77,78] helped hospitals anticipate bottlenecks during pandemic waves. These techniques proved particularly valuable for pharmacies [61] and blood banks [32] managing just-in-time inventory systems. Finally, ref. [79] systematically reviews ED visit prediction models, validating ARIMA’s superiority for short-term forecasts, while [80,81] proposes hybrid methods for medical service demand forecasting.

Additionally, the limited allocation of resources during pandemics led to a rigorous ethical analysis. Operational guidelines from Maryland [82] and Rochester [83] established transparent protocols for ventilator triage, while empirical studies [73,84] assessed algorithmic bias in bed allocation. Debates over frailty scoring [85] and ECMO prioritization [86,87] revealed tensions between utilitarian efficiency and equity principles. Legal analyses [88] of state-level crisis plans identified gaps in disability protections, and ICU triage simulations [63] demonstrated how implicit biases could affect survival predictions. The “reserve system” concept [89] offered a compromise by stratifying access criteria for different patient groups, though implementation challenges remained, as discussed in [87,90,91].

Finally, long-term strategies for hospital resilience integrate demand forecasting, capacity planning, and cross-sector coordination. Regional dashboards [36,92,93] enabled real-time bed monitoring across France, while discrete-time simulations [94] projected decade-long demand for Belgian hospitals. In ref. [95] a supply-demand gap analysis in Dubai hospitals (2018–2030) provided a template for long-term capacity planning in growing populations. Comparative studies of supply chains [30,96] identified vulnerabilities in PPE logistics, and pediatric mobile clinic models [97] demonstrated adaptive service delivery for vulnerable populations. The COVID-19 experience underscored the need for: (i) flexible staffing pools [52], (ii) scalable infrastructure [98], and (iii) embedded ethical review processes [82,99]. Moving forward, integrating these lessons into national preparedness plans [13,88] will be crucial for mitigating the impact of future health crises, as highlighted in [24,37].

### 3.2. IT Algorithms

The COVID-19 pandemic exposed critical weaknesses in healthcare systems worldwide, particularly in their ability to respond to sudden surges in patient demand. As global health systems continue to face the dual challenges of recurrent outbreaks and long-term demand variability, accurate forecasting of hospital surge capacity remains essential. A central objective of this systematic review was to consolidate findings from the literature regarding methodologies and tools used to anticipate hospital bed occupancy and resource requirements, with a focus on data-driven, statistical, and machine learning approaches, and their impact on operational planning and policy development.

Studies consistently emphasize the necessity of forecasting hospital bed demand, especially during infectious disease outbreaks, to prevent care bottlenecks [42]. Tools like the Bed Occupancy Management Planning System (BOMPS) and predictive models such as ARIMA, exponential smoothing, and neural networks have been widely adopted [77]. These techniques demonstrate high predictive accuracy when tailored to hospital-specific catchment areas, outperforming generic national-level forecasts [42,81].

A key insight is the superiority of localized models. For instance, the J-IDEA pandemic planner developed by [98] enables scenario-based modeling of hospital interventions, offering tangible support to health administrators making real-time decisions. The model incorporates both demand-side strategies (e.g., elective surgery cancellations) and supply-side interventions (e.g., field hospital construction, workforce redeployment) to simulate surge response strategies [98]. This integrated modeling is further supported by data from tools like CHIME and the CDC COVID-19 Surge Tool [100], which inform hospital administrators on how to adjust capacity based on projected need.

Demand prediction has also evolved through the application of machine learning models, which accommodate complex nonlinear patterns in patient flow data. Ref. [77] demonstrated the potential of K-SVR (Kernel Support Vector Regression) in achieving low mean absolute error rates using structured electronic health record data, highlighting the strength of machine learning in forecasting short-term inpatient demand. Other models, such as those in ref. [101], used recurrent neural networks to estimate bed occupancy with high precision, especially for forecasts within a 1–2 day horizon.

Notably, forecasting tools are most accurate when they consider local epidemiological dynamics and healthcare infrastructure constraints. For example, differences in hospital catchment area definitions, staffing availability, and regional incidence trends can drastically influence the reliability of models [43]. Furthermore, ethical considerations and triage policies, such as those discussed in ref. [102], underscore the need for models that also support just allocation of care resources under scarcity.

Beyond prediction, case studies also explored the operational implications of forecast results. For instance, capacity management through patient mix optimization has been a focus area, where mathematical models optimize elective surgery scheduling and resource utilization [103]. Similarly, demand estimation has influenced emergency department staffing, as illustrated in studies using simulation and stochastic modeling approaches [81]. Dashboards and digital interfaces allowing hospital-specific model customization, as done in Germany’s on-demand forecasting platforms, represent a promising future direction [43].

Moreover, challenges remain in the adoption of these tools. Many hospitals, particularly in low-resource settings, lack the digital infrastructure or analytical capacity to operationalize complex models. Hence, tools with minimal data requirements and high interpretability are favored, as shown in ref. [104], where mixed-integer programming enabled tractable solutions for case mix planning under uncertainty.

Overall, this review indicates a trend toward hybrid modeling strategies that combine historical data, real-time surveillance inputs, and hospital-specific operational parameters to optimize hospital response during demand surges. There is a growing consensus that forecasting models should be embedded in routine hospital operations to foster preparedness for future crises and manage fluctuating patient inflows under normal conditions.

### 3.3. Ethical Dimension

The COVID-19 pandemic significantly disrupted global health systems, highlighting the importance of hospital surge capacity and ethical resource allocation. In this systematic review, we synthesize evidence from 27 key studies and policy reports across North America, Europe, Australia and Asia to understand how healthcare systems evaluate and prepare for surge events, with an emphasis on organizational readiness, workforce resilience, ethical classification protocols, and competing equity imperatives.

Hospital surge capacity encompasses the ability of healthcare facilities to rapidly scale resources—staff, space, and supplies—in response to a sudden increase in patient volume. This includes not only infrastructural elements, but also operational systems and leadership structures that guide decision-making during crises. Ref. [105] argues that crisis standards of care must be developed regionally to ensure equitable distribution of scarce resources and reduce interinstitutional disparities in access. A regionalised triage mechanism, such as that adopted by the Washington State Disaster Clinical Advisory Committee, serves as a coordination tool to balance load and avoid overwhelming any single facility. Ethical frameworks are integral to surge response planning. The pandemic underscored the need for transparent, equitable and inclusive decision-making structures. Ref. [106] emphasizes that resource allocation decisions must be underpinned by procedural fairness, including the separation of triage duties from direct patient care to protect clinicians and uphold objectivity. Similarly, authors in ref. [107] underscore the importance of pediatric-specific considerations in crisis care, noting that the application of illness severity scoring systems must not exacerbate existing health inequities.

Triage protocols, such as SOFA and PELOD scores, have been widely adopted to guide ICU admissions and resource rationing during emergencies. However, multiple sources caution that prognostic scoring systems can unintentionally establish disparities if used without contextualization. For example, ref. [108] critiques “exclusive survivalism”, a utilitarian approach to assessing that prioritizes saved lives above all else, as it risks compounding systemic injustices by prioritizing marginalized groups with a lower baseline life expectancy. Evaluation of organisational readiness was another recurring theme. In ref. [102] the authors proposed a model that includes six core capabilities: monitoring hospital occupancy, staff availability, PPE and ventilator supply chains, communication structures, command and control infrastructure, and rapid policy adaptation. In a parallel discussion, the Hastings Center [108] highlights the moral complexity of balancing individual rights against public good, advocating for inclusive stakeholder engagement when developing allocation policies.

Several case studies illustrate the operationalisation of surge preparedness. For instance, the Alberta health system’s staged response approach enabled the dynamic reallocation of ICU beds and staff based on infection modelling and real-time capacity assessments. Meanwhile, European policy guidance from SIAARTI and the European Society of Intensive Care Medicine provides operational protocols for critical care triage, highlighting criteria such as expected survival and reversibility of disease [109]. Beyond logistics, this review foregrounds the importance of aligning surge strategies with equity. In the UK context, authors of [110] argue that the NHS’s shift from horizontal equity (equal access for equal clinical need) to vertical equity (unequal treatment to equalise outcomes) has unintentionally disadvantaged older rural populations. NHS funding formulas that favour younger, deprived urban areas often neglect the high disease burden in ageing communities, creating systematic under-provision in regions most vulnerable during a pandemic.

This tension is mirrored in U.S. literature, where federal and state triage protocols have struggled to balance efficiency and justice. Tools such as ventilator allocation frameworks often incorporate clinical prediction scores that correlate with social disadvantage, thereby exacerbating disparities unless adjusted for sociodemographic context. As the authors in ref. [106] stress, planning must incorporate mechanisms to review and revise decisions in light of new evidence and ensure that vulnerable populations are not structurally excluded from lifesaving care. Workforce dynamics also featured prominently. Shortages in frontline personnel due to illness, burnout, or redeployment were reported across settings. Several studies proposed the use of flexible staffing models and telemedicine as mitigation strategies. Notably, the use of “team-based triage” helped distribute decision burdens and reduce moral distress among clinicians, as described in [107].

Additionally, the role of temporary facilities—ranging from field hospitals to repurposed event centres—was explored. While these expansions increased physical capacity, their effectiveness was constrained by workforce and equipment limitations. Ref. [111] recommends that such facilities be integrated into central coordination platforms for resource visibility and efficient referral. From a policy perspective, guidance from the CDC and WHO was critical in shaping institutional response. However, the actual implementation of these guidelines varied widely. The importance of inter-hospital and cross-jurisdictional data sharing emerged as a key determinant of successful surge response, particularly when it came to reallocating scarce resources like ventilators or ECMO capacity. On the societal level, the review also considers the ethics of communication and trust-building. Transparency in decision-making—such as explaining triage policies to the public and involving communities in planning—was repeatedly cited as essential for maintaining legitimacy. According to ref. [112] the authors called for deliberate efforts to confront disability-based discrimination, warning that triage systems that overly rely on quality-of-life assumptions may disproportionately disadvantage disabled individuals.

### 3.4. Case Studies from Different Countries

The COVID-19 pandemic has revealed both the strengths and the vulnerabilities of healthcare systems worldwide, catalyzing a surge in research on preparedness, system resilience, and adaptive service delivery. In response to the unprecedented global health crisis, numerous countries implemented diverse strategies to meet evolving demands in patient care, workforce capacity, and public health coordination. These case studies reveal that healthcare resilience—defined as a system’s ability to absorb shocks, adapt to pressure, and maintain essential services—is a multidimensional construct shaped by socio-economic structures, governance models, workforce management, and infrastructural readiness. Key attributes influencing healthcare system responsiveness included the scalability of intensive care unit (ICU) capacity, strategic workforce planning, the integration of digital technologies, and the equity of resource distribution. Countries with proactive simulation-based planning, robust occupational health structures, and effective inter-agency coordination were often better positioned to manage patient surge capacity.

From an epidemiological forecasting and resource planning perspective, several studies employed sophisticated modelling tools to predict future healthcare demands and assess system capacity. In Australia, authors in ref. [113] conducted a population-based projection study focusing on the trends and future projections of Permanent Residential Aged Care (PRAC) services. Their findings highlighted a decline in PRAC admission rates over a decade, with projections indicating continued reductions per capita, despite an expected increase in the total number of admissions due to population ageing through 2037. In Belgium, the ProMoBed was developed [94], a dual-component model that combines extrapolation and simulation to forecast inpatient bed requirements. The model adjusted for demographic changes and pathologies and used Shapley value analysis to distinguish causal drivers of inpatient demand. Forecasting also extended to workforce planning. In Ontario, Canada, the study of [114] projected substantial shortages in full-time equivalent personal support workers and nurses in the long-term care sector by 2035. To address strategic midwifery workforce development, authors in [115] applied the UK’s Robust Workforce Planning Framework in Belgium, co-developing policy scenarios through surveys and stakeholder workshops. Similar workforce challenges were reported in Japan, Korea, and the USA, where ageing populations and provider burnout prompted governments to reconsider health human resource policies and shift towards integrated care models.

Crisis preparedness and surge capacity management emerged as dominant themes. According to ref. [53,116], a two-part assessment of ICU capacity in Australia highlighted workforce limitations as the main constraint during the pandemic. Although ventilator availability was sufficient, workforce limitations, especially among ICU nurses, constrained surge responsiveness. In addition, the authors in ref. [117] extended this analysis to air medical logistics via the Royal Flying Doctor Service, developing a discrete event simulation model to estimate aircraft and staffing needs under increased COVID-19-related demands. Parallel findings from Germany, France, and Spain identified similar gaps in trained staff availability as a limiting factor, despite adequate physical infrastructure. Adaptive service delivery models were exemplified in the oncology sector. Ref. [118] examined operational adjustments in a radiation oncology department in Victoria, Australia. These included split shifts, remote work, and digital communication, which were met with high staff satisfaction and readiness to continue post-pandemic, especially among staff with caregiving responsibilities. Ireland and the Netherlands implemented similar telehealth expansion strategies, while Sweden and New Zealand adopted hybrid models combining digital and in-person care based on epidemiological risk.

In Canada, several studies shed light on broader policy and coordination mechanisms. Ref. [119] presented an environmental scan of 470 pandemic-era workforce strategies implemented nationwide. These included telehealth expansion, role adaptation, licensure flexibility, mental health initiatives, and return-to-practice programs. In ref. [120], the authors highlighted regional coordination through the Toronto Academic Health Science Network (TAHSN), a consortium of 14 healthcare organizations aligning their Occupational Health and Safety (OHS) practices to respond to burnout and inconsistency in safety protocols. Comparable inter-organisational collaborations were observed in Portugal and Italy, where cross-hospital coordination reduced duplication and improved capacity management. In humanitarian and low-resource settings, the pandemic’s burden was particularly severe. In Bangladesh, authors of ref. [52] modelled potential COVID-19 outbreaks in the Kutupalong-Balukhali refugee camp using a SEIR transmission framework. Their scenarios projected rapid spread and overwhelming hospitalisation needs, far exceeding the existing healthcare capacity. Similar concerns were documented in Ghana [121], Uganda [122], and India [123], where underfunded health systems faced compounding barriers including lack of diagnostics, personal protective equipment, and vaccine inequities. Local adaptations included the deployment of mobile clinics in Uganda and task-shifting policies in India to expand frontline service coverage.

In Chile, ref. [70] proposed a bi-objective stochastic optimisation model for improving inpatient admission planning. Their approach, validated with real hospital data, balanced service cost against resource utilisation under uncertain patient inflows. Moreover, in China, ref. [124] assessed equity and efficiency in health resource allocation across Sichuan Province. Their analysis, using Theil index, HRAD, and data envelopment analysis, revealed persistent inequalities across economic zones, despite overall growth in health resources. These equity assessments find resonance in analyses from Colombia [125] and Iran [49,126], which reported stark rural-urban disparities in health system capacity and service continuity. From a disaster preparedness standpoint, Austria provided further insights. In ref. [63] the authors evaluated mass casualty response planning using discrete event simulation and regression-based scenario analysis in a Vienna hospital. Their findings stressed that structural redesign, optimal staff-mix, and increased radiology and rotation staff were more impactful than equipment scale-up alone. The UAE [95] and Saudi Arabia [127,128] also adopted pre-emptive simulation exercises to model pandemic responses and optimise cross-agency coordination, showing that structured emergency preparedness could reduce operational delays.

Economic evaluation frameworks also played a central role in resource prioritisation debates. Ref. [129] critiqued Australia’s unusually high healthcare discount rate, suggesting it undermines the value of preventive services and biases investment decisions toward short-term gains. Similar critiques were raised in the USA [130] and UK [68], where emergency spending often lacked long-term sustainability frameworks, prompting calls for integrated economic and public health planning post-pandemic. Canada’s surgical system was another focus of modelling efforts. The authors in ref. [131] applied ARIMA models to predict orthopedic and ophthalmologic surgical volumes in Manitoba through 2027. Their findings showed growing demand, especially in clinic-based settings, highlighting the need for comprehensive integration of administrative data to guide capacity planning. Comparable backlog forecasting models were developed in Spain and Korea, helping to stratify waiting lists and inform surgical triage protocols.

From the reviewed literature, it is evident that while high-income countries placed strong emphasis on epidemiological forecasting, digital health transformation, and workforce flexibility, the implementation of these strategies was often constrained by systemic bottlenecks, including staffing shortages and fragmented policy coordination. In contrast, low-resource settings and refugee-hosting regions faced fundamentally different constraints, requiring bespoke, context-sensitive solutions tailored to their operational realities and infrastructural limitations. Despite the diversity of contexts, a unifying theme emerges: the need for adaptive, equitable, and forward-looking health system strategies. These strategies must transcend reactive crisis response to enable sustained resilience, informed by integrated planning, inclusive governance, and investment in both human and technological capacities.

## 4. Policies and Lessons

The COVID-19 pandemic exposed fundamental weaknesses in global healthcare systems’ ability to respond to sudden surges in demand [18,31], prompting an urgent reevaluation of workforce strategies and resource allocation frameworks. The ARRAS framework (Anticipate, Recruit, Retain, Adapt, Sustain) proposed by [52] emerged as a particularly valuable model, especially for low- and middle-income countries. Their research in Bangladesh, Nepal, and Sri Lanka demonstrated how pre-crisis planning, combined with adaptive measures like telemedicine deployment and workforce reskilling [132], could maintain essential health services despite severe budget constraints. However, the study also revealed persistent systemic challenges, including fragmented data systems and inadequate funding mechanisms [27], that limited the potential effectiveness of these interventions.

These findings align closely with the broader observations of [119], whose comprehensive scoping review of 37 studies identified three critical components for effective crisis response: rapid workforce expansion through innovative recruitment strategies [133], enhanced role flexibility via cross-training and scope-of-practice modifications [54], and comprehensive staff support systems to address the psychological toll of prolonged crisis conditions [134]. The strain on intensive care capacity during the pandemic prompted innovative approaches to surge management that warrant careful examination [44,47]. Ref. [135] provided detailed evidence-based recommendations for mass critical care situations, emphasizing tiered staffing models that incorporated non-ICU specialists [69], regional load-balancing through coordinated patient redistribution [30], and the strategic use of tele-ICU technologies to extend specialist coverage [36].

These theoretical frameworks found practical application in settings like Australia, where [54] documented the successful deployment of ICU-trained physiotherapists in expanded team-based care models. While this approach effectively addressed immediate staffing shortages [120], qualitative feedback from frontline providers highlighted unintended consequences, particularly regarding role ambiguity and accelerated burnout when expanded responsibilities were not accompanied by clear boundaries or additional support [134]. The tension between crisis-driven innovation and sustainable practice emerged as a recurring theme across multiple studies [136], with [133]’s analysis of the Canadian response providing particularly stark examples of temporary measures that showed promise but failed to transition into enduring system improvements [119].

Telehealth adoption during the pandemic served a dual purpose, functioning both as a demand management tool and an early warning system [137]. The longitudinal study by [48] yielded compelling evidence that COVID-19-related telehealth inquiries could predict emergency department surges with remarkable accuracy, demonstrating an 85% correlation between telehealth volume and subsequent ED visits. This predictive capacity, when combined with the machine learning approaches for EMS demand forecasting described by [18,28], suggested new possibilities for proactive resource allocation [77]. However, the implementation of triage systems like England’s “total triage” model, analyzed by [138], revealed complex trade-offs. While such systems demonstrated potential to reduce unnecessary ED utilization by 15–20% [74], they also introduced new equity concerns, particularly for vulnerable populations with limited digital literacy or access [110]. Furthermore, the increased administrative burden on clinical staff, often without corresponding increases in support resources [139], created additional stress points in already strained systems [99].

Ethical dilemmas surrounding resource allocation emerged as one of the most profound challenges of the pandemic response [140]. The work of [139] provided crucial insights into the psychological impact on healthcare providers forced to make rationing decisions, identifying moral distress, guilt, and system-induced shame as significant threats to workforce sustainability [134]. Their recommended mitigation strategies, including transparent triage protocols [135], dedicated rationing teams [46], and structured psychological support programs [120], offered a framework for preserving staff well-being during crisis conditions. These ethical considerations intersected with broader questions of health equity [93], where ref. [110] critique of England’s NHS funding allocations revealed how philosophical shifts toward outcome-based equity measures had inadvertently disadvantaged aging and rural populations [27]. In contrast, the regional health conference model described by [141] demonstrated how localized, data-informed decision-making could balance efficiency with equity considerations [80].

Synthesis of this extensive body of research [119,142] suggests several critical pathways for strengthening health system resilience. Workforce strategies must evolve beyond temporary crisis measures to institutionalize flexible licensure frameworks and cross-training protocols while investing in comprehensive staff support systems [52,133]. Predictive analytics, particularly those integrating telehealth data with advanced modeling techniques [33,48], should be prioritized for resource allocation decision-making [143]. Ethical frameworks for crisis response require careful balancing of transparency, equity, and psychological support for decision-makers [139,140]. Perhaps most fundamentally, the pandemic underscored the necessity of shifting from reactive to proactive surge planning [47], with [142]’s resilience framework providing a comprehensive approach encompassing governance, financing, and service delivery adaptations [136].

The consistent gap in rigorous outcome evaluation across studies [119], noted by [42], highlights an urgent need for embedded research mechanisms to assess the real-world effectiveness of implemented strategies [143]. These collective findings pave the way for healthcare systems [136], where the lessons of the pandemic must inform not just preparation for future crises, but the fundamental restructuring of how health workforces are organized [133], resources are allocated [27], and equity is operationalized [110]. The transition from temporary adaptations to sustainable reforms emerges as the central challenge [119], requiring coordinated action across the policy, practice, and research domains [142]. As [136] compellingly argue, the post-pandemic era demands nothing less than a reimagining of public health infrastructure [141], with resilience, equity, and adaptability as foundational principles rather than crisis-driven afterthoughts [142]. The studies reviewed collectively provide both a warning about the costs of unpreparedness and a roadmap for building health systems capable of withstanding future shocks while delivering on their fundamental mission of equitable care [27,93].

## 5. Discussion

**Figure 2:**
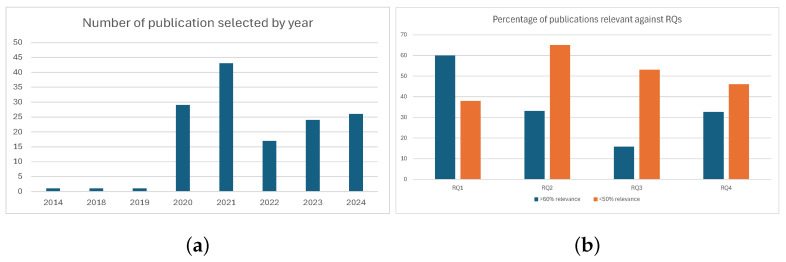
Search results for the 142 selected papers. (**a**) The selected 142 articles grouped according to the year of publication. (**b**) Relevance of articles addressed against each RQs, as outlined in Table 1.

The COVID-19 pandemic has underscored critical weaknesses in healthcare systems worldwide, particularly with regard to their capacity to absorb surges in patient demand and maintain continuity of care. The results presented in this systematic review illuminate several interrelated domains—hospital resource planning, forecasting methodologies, workforce readiness, and ethical governance—that together shape healthcare operational system resilience are depicted in Figure 2. Across these domains, a shared emphasis emerges: the imperative for anticipatory, adaptive, and ethically grounded strategies that are contextually tailored and operationally feasible. The quality and consistency of evidence varied considerably across studies, reflecting differences in methodological design. Simulation models and predictive algorithms often demonstrated strong internal validity but limited generalizability, whereas empirical case studies provided rich contextual insights but were sometimes limited in scope. Policy evaluations contributed valuable lessons but were often context-dependent, with applicability constrained by local governance structures. Contextual factors also shaped the feasibility of surge strategies. High-income countries often leveraged digital forecasting tools and flexible staffing policies, whereas low- and middle-income settings emphasized community-based care, mobile clinics, or ad hoc workforce redeployment. These variations underscore the importance of tailoring surge planning frameworks to local resource availability and governance models.

A dominant theme throughout the literature is the centrality of predictive analytics in anticipating hospital resource needs. Studies leveraging machine learning models, hybrid statistical approaches, and grey systems theory demonstrated significant improvements in the accuracy of forecasts for bed occupancy, ICU demand, and emergency service usage. Particularly effective were models integrating local epidemiological data with operational parameters such as catchment demographics, staffing constraints, and elective care scheduling. These localized tools consistently outperformed national-level predictions and proved instrumental in optimizing resource deployment. Nevertheless, the operational translation of these forecasts was not without challenges, particularly in contexts lacking digital infrastructure or analytic capacity. In such settings, simplified models emphasizing interpretability and real-time data integration emerged as pragmatic alternatives.

Workforce adaptability surfaced as another pillar of resilience. The ARRAS framework and similar models proposed in South Asia, Canada, and Australia underscore the importance of sustained investment in flexible staffing models, cross-training programs, and psychological support systems. Notably, successful strategies combined short-term redeployment with long-term role evolution, including the use of telehealth, task-shifting, and scope-of-practice expansions. However, these approaches frequently encountered implementation bottlenecks, including regulatory inertia, fragmented human resource governance, and a lack of institutional frameworks to support staff well-being. Feedback from frontline workers, particularly in Canada and Australia, revealed that innovative staffing solutions introduced under crisis conditions were often poorly resourced or ambiguously defined, leading to role ambiguity and increased burnout.

The ethical allocation of scarce resources emerged as both a moral and operational challenge. The review highlights the need for transparent, procedurally fair, and inclusive triage protocols that are sensitive to local inequities and systemic biases. Tools such as SOFA and PELOD scores provided structured approaches to ICU triage but risked reinforcing pre-existing disparities unless contextualized appropriately. In the United States and United Kingdom, debates over vertical versus horizontal equity in funding allocation underscored the policy implications of ethical frameworks. In particular, the disproportionate burden borne by rural, aging, or socioeconomically disadvantaged populations during the pandemic revealed the unintended consequences of outcome-based equity models. These findings support calls for ethical frameworks that embed equity assessments into preparedness planning and triage decision-making.

Case studies from across 27 countries further illustrate the diverse strategies employed to navigate pandemic-induced pressures. High-income countries like Australia, Belgium, and Canada emphasized forecasting tools, digital transformation, and workforce flexibility. For example, the ProMoBed model in Belgium and aged care demand forecasting in Australia exemplified advanced simulation-driven approaches. However, systemic constraints, such as workforce shortages, inadequate coordination across agencies, and inconsistent policy implementation, often limited the impact of these innovations. In contrast, low- and middle-income countries such as Bangladesh, Ghana, and India adopted bespoke interventions, leveraging mobile clinics, localised workforce policies, and community-based planning to expand service coverage. These approaches were frequently shaped by infrastructural deficits and epidemiological burdens but underscored the value of contextually relevant, bottom-up solutions.

Cross-national analyses also revealed common points of failure and opportunity. Countries with pre-existing coordination frameworks, integrated data systems, and decentralised planning capacities, such as Portugal, the Netherlands, and Sweden, were generally more agile in responding to demand surges. Conversely, fragmented systems struggled with timely resource reallocation and staff deployment. Ethical governance, real-time data dashboards, and modular infrastructure design were recurring features of successful responses. Moreover, the operationalisation of preparedness plans often hinged on intersectoral coordination and community engagement, highlighting the need for a whole-of-system approach to resilience building.

Taken together, the reviewed studies emphasize that hospital surge preparedness is not merely a function of physical capacity but rather the intersection of predictive capability, workforce sustainability, ethical clarity, and adaptive governance. While technical innovations, particularly in forecasting and optimization, offer substantial potential, their effectiveness is contingent on institutional readiness, equitable policy frameworks, and inclusive implementation. As health systems transition from crisis response to long-term reform, embedding these multidimensional insights into national and regional preparedness strategies will be critical to future resilience. Future research should prioritize the development of standardized and comparable metrics for assessing organizational surge readiness, rigorous comparative evaluations of policy effectiveness across jurisdictions, and integration of ethical considerations into predictive models and workforce planning tools. Methodologically, there is also a need for mixed-methods designs that combine quantitative forecasting with qualitative insights on workforce resilience and governance dynamics.

## 6. Conclusions

In this systematic review, we extensively presented the current literature regarding the optimization of emergency response in hospitals through surge capacity planning and crisis resource management. More specifically, we selected a total of 142 articles for inclusion from an initial pool of 392, presenting key advancements in resource allocation, workforce resilience, and IT-based forecasting tools. Moreover, best practices identified in the literature, including predictive modeling, ethical protocols, and adaptive infrastructure, should be promoted among healthcare stakeholders, including clinicians, paramedics, administrators, and policymakers. Despite the extensive research on emergency preparedness, gaps remain in standardizing metrics for organizational readiness and integrating ethical considerations into crisis planning. Future work should focus on developing systematic frameworks to harmonize research findings and evaluate the effectiveness of surge strategies in different healthcare settings. In addition, objective benchmarks are needed to assess hospital resilience and facilitate cross-institutional collaboration. Taken together, the findings emphasize the interdependence of technical forecasting, operational planning, and ethical governance in hospital surge preparedness. While our synthesis was primarily descriptive, the thematic overlaps observed suggest that integrative frameworks linking these dimensions will be essential for guiding both future research and practical policy development.

## Figures and Tables

**Table 1:** Research questions and background considered for investigation in this systematic review.

Thematic Focus Area	Research Questions	Investigation Scope
Hospital Resource Management	**RQ1:** How do demand forecasting models optimize allocation of beds, staff, and equipment during surge events?	Effectiveness of predictive algorithms, inventory management systems, and real-time resource tracking
IT & Algorithmic Solutions	**RQ2**: What role do IT and AI/ML algorithms play in predicting patient surges and automating emergency responses?	Evaluation of decision-support systems, predictive modeling accuracy, and integration with hospital workflows
Ethical Considerations	**RQ3:** What ethical challenges emerge in resource rationing and priority-setting during hospital crises?	Analysis of protocols, equity in access to care, and staff moral distress
Policy & Lessons	**RQ4:** How effective are current emergency management policies in ensuring healthcare system resilience?	Comparative evaluation of national/institutional policies, regulatory frameworks, and compliance measures

**Table 2:** Scoring framework for article evaluation.

Criterion	Description	Score Range	Evaluation Guidance
Relevance to research questions	Degree to which the study addressed at least one of the predefined RQs	1–5	1 = marginal relevance, 5 = highly relevant
Methodological rigor	Quality of design (e.g., empirical data, systematic methodology)	1–5	1 = low rigor, 5 = high rigor
Contextual applicability	Extent to which findings are applicable to hospital surge/emergency settings	1–5	1 = limited relevance, 5 = direct relevance
Contribution to thematic domain	Fit within surge capacity, resource allocation, ethics, or policy	1–5	1 = minor contribution, 5 = substantial contribution
Clarity of reporting	Transparency and completeness of study reporting	1–5	1 = poorly reported, 5 = well-reported

## Data Availability

No new data were created or analyzed in this study. Data sharing is not applicable to this article.
